# Opium Addiction Increases Interleukin 1 Receptor Antagonist (IL-1Ra) in the Coronary Artery Disease Patients

**DOI:** 10.1371/journal.pone.0044939

**Published:** 2012-09-19

**Authors:** Habibollah Saadat, Seyed Ali Ziai, Maryam Ghanemnia, Mohammad Hasan Namazi, Morteza Safi, Hosein Vakili, Ali Dabbagh, Omid Gholami

**Affiliations:** 1 Cardiovascular Research Center, Shahid Beheshti University of Medical Sciences, Tehran, Iran; 2 Pharmacology Department, School of Medicine, Shahid Beheshti University of Medical Sciences, Evin, Tehran, Iran; 3 Anesthesiology Research Center, Shahid Beheshti University of Medical Sciences, Tehran, Iran; Sapienza University of Rome, Italy

## Abstract

**Background:**

There is evidence that opium addiction has immunosuppressant effects. Coronary artery disease (CAD) is a condition resulted from atherosclerosis which is dependent on the immune response.

**Purpose:**

To evaluate plasma levels of interleukin-6 and interleukin-1Ra in 30 patients with three-vessel coronary artery disease, ejection fraction of more than 35% and to evaluate their changes after prognostic treadmill test in 15 opium addicted and 15 non-addicted patients.

**Methods:**

The participants underwent prognostic treadmill test and plasma levels of interleukin-6 (IL-6) and interleukin-1Ra (IL-1Ra) were evaluated with ELISA method before, just after and 4 hours after the test.

**Results:**

IL-1Ra (2183 pg/ml) tended to decrease over time in the opium addicted group (1372 pg/ml after prognostic treadmill test and 1034 pg/ml 4 hours after that), although such decrease did not reach the statistical significance. IL-1Ra levels were significantly higher in opium addicted than in non addicted patients. Opium addiction had no significant effect on IL-6 changes.

**Conclusion:**

Consumption of opium in CAD patients is associated with higher IL-1Ra levels.

## Introduction

Atherosclerosis is a chronic inflammatory process, which is characterized by leukocyte proliferation and migration of smooth muscle cells, matrix formation, revascularization and cytokine formation [1]. Recent researches suggest that inflammatory phenomenon, at the site of atherosclerotic plaque are major determinants of the progression of the disease and many circulating inflammatory chemicals such as cytokines and acute phase proteins have been studied in patients affected by coronary disease [1], [2], [3]. Baseline plasma levels of IL-1Ra (interlukin-1 receptor antagonist) are increased in patients with atherosclerosis [3]. IL-1Ra and IL-6 are significantly increased in coronary artery disease (CAD) patients compared with healthy individuals [4], [5].

Opium addiction is a major social problem. There is a belief among people that opium has protective effect on cardiovascular disease and few studies available about its effect on atherosclerosis show controversies [6], [7], [8]. Chronic opium consumption may lead to decreasing lymphocyte proliferation and affects inflammatory mediators. Morphine in rats’ peritoneal macrophages enhanced interleukin (IL)-12 and tumor necrosis factor alpha (TNF-α) induced by lipopolysaccharide [9]. Chronic morphine treatment in mice resulted in a significantly higher (two- to threefold) activation of macrophage TNF-α and IL-1β synthesis occurred with chronic morphine treatment [10]. Within 2 h after heroin administration, proliferative responses to alloantigens and the production of IL-1β, IFN-γ, IL-12 and NO were enhanced significantly in mice spleen cells. In contrast, production of anti-inflammatory cytokines IL-4 and IL-10 was at the same time rather decreased [11].

Chronic morphine treatment in vivo and in vitro decreases IL-2 and IFN-γ (T helper 1) protein levels and increases IL-4 and IL-5 (T helper 2) protein levels in a time-dependent manner [12].

Fiotti et al showed changes of the main pro inflammatory cytokines TNF-α, IL-1β, IL-6, their soluble receptor antagonist and a variety of inflammatory markers in patients with peripheral arterial disease (PAD) after treadmill test [3]. In this study we determined IL-6 and IL-1Ra changes after prognostic treadmill test (as a stressor maneuver) to compare the levels of selected plasma cytokine levels between opium-addicted and non-opium addicted male patients with coronary artery disease.

**Figure 1:**
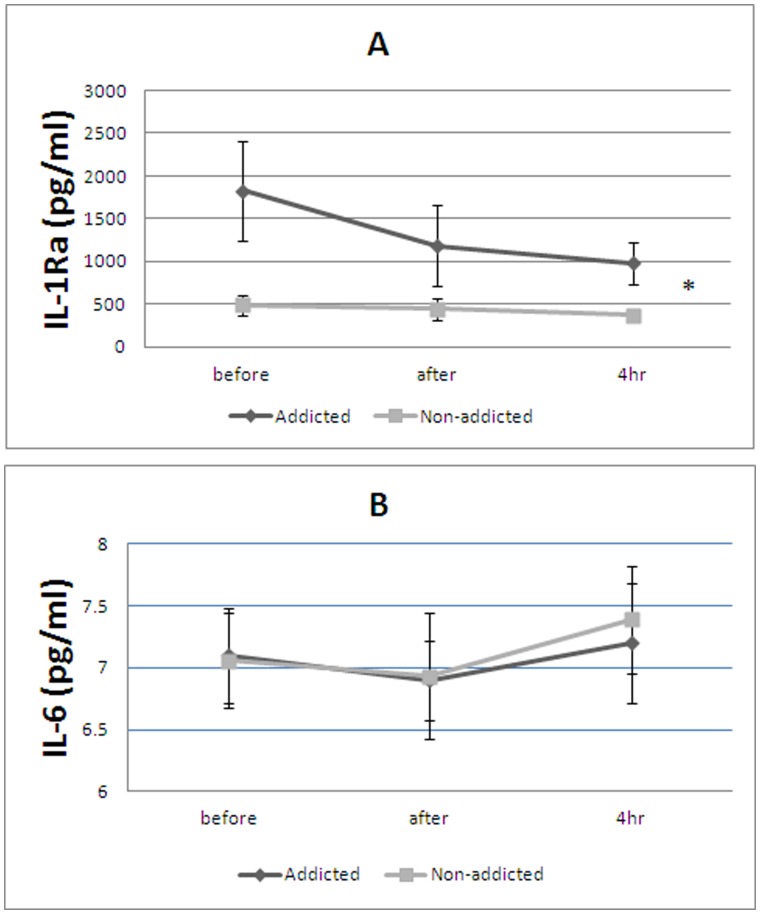
Effect of treadmill test on IL-1Ra (A) and IL-6 (B) in the opium-addicted and non-addicted patients. Each point represents mean ± SE. of mean (*p<0.05 between groups based on repeated measurement method analysis).

## Materials and Methods

### Patients and Treadmill Test

Fifteen patients with only opium addiction (raw opium inhalation) and fifteen non- addicted patients suffering from three-vessel coronary disease documented by coronary angiography and ejection fraction (EF) >35% were studied. All patients were men, current smoker, and mean age was 54.7±1.7.

Notably, vascular problems, and acute coronary syndrome from 2 months before, infection, malignancy, hepatic disease, renal failure (creatinine >1.5 mg/dl), diabetes mellitus, history of pacemaker implantation, history of major trauma and surgery were criteria for exclusion from the study. Careful history and physical examination were carried out and medications were not discontinued. These two groups underwent a treadmill test with prognostic protocol. During the test, continuous ECG monitoring was performed in all patients. The two groups were matched regarding dyslipidemia and hypertension. All of the patients received aspirin, statin, angiotensin converting enzyme inhibitor, beta receptor blocker, and nitrates.

**Table 1:** IL-6 and IL-1Ra tracks before, just after and 4 hours following treadmill test in the opium-addicted and non-addicted 3-vessel CAD patients.

Cytokine		Before	After	4 hr after
IL-6 (pg/ml)	Opium-addicted	7.04 (1.44)	6.8 (1.02)	6.71 (1.35)
	Non-addicted	6.88 (1.35)	7.04 (1.48)	7.79 (1.29)
IL-1Ra (pg/ml)	Opium-addicted	2183.0 (1894)	1372.0 (1974)	1034.3 (891.7)
	Non-addicted	520.3 (418.43)	452.9 (490.43)	353.08 (241.33)

Values are represented as mean (STD. Deviation of mean).

**Table 2:** Laboratory parameters measured among the opium-addicted and non-addicted patients.

	Opium-addicted	Non-addicted	P value
Ejection Fraction (%)	50.80 (5.63)	49.60 (8.17)	0.644
Hemoglobin (g/dL)	13.87 (0.83)	14.53 (1.64)	0.176
AST(U/L)	16.87 (4.49)	16.13 (4.01)	0.641
Urea(mg/dL)	24.39 (8.92)	23.33 (7.18)	0.723
ESR (mm/h)	16.40 (5.34)	15.07 (5.23)	0.495
ALT (U/L)	16.47 (5.10)	18.20 (4.28)	0.322
Creatinine (mg/dL)	0.87 (0.35)	0.93 (0.25)	0.559
LDL (mg/dL)	136.20 (24.21)	123.80 (11.19)	0.087

Values are expressed as mean (STD. Deviation of mean). P values are calculated based on ANOVA test.

### Blood Samples

Patients were kept fasting for 8 hours before the test. Blood samples were obtained from all patients with 16 G needle and collected in syringes. The blood was immediately placed on melting ice until it was centrifuged at 2000 g at 4°C for 30 min. the plasma was kept in −20°C until it was analyzed.

Blood samples in the 2 groups were obtained before, immediately after treadmill test and 4 hours later in all patients.

ESR, ALT, AST, Hgb, Creatinine, Urea and LDL were measured in all patients before treadmill prognostic test.

### Cytokines Analysis

Cytokines were determined by IL-6 and IL-1Ra ELISA kits (R&D system Minneapolis, MN). The assays employed the quantitative enzyme immunoassay technique.

### Statistical Analysis

Correlations among inflammatory markers and laboratory findings were analyzed by Spearman’s rank correlation coefficient. Repeated measurement ANOVA was conducted within time series between two addicted and non-addicted groups. P values less than 0.05 were considered to indicate statistical significance.

### Medical Ethics

This study was approved by Medical Ethics Council of Shahid Beheshti University of Medical Sciences, Tehran, Iran (Research number # 17562/13 on 9 September 2007). Written informed consent was obtained from all participants.

## Results

### Cytokines

Values of plasma levels of cytokines in opium addicted and non-addicted 3-vessel disease patients at three times: before, after, and 4 hours after treadmill test are reported in table 1 and figure1.

IL-1Ra in the addicted patients was significantly higher than non-addicted patients (Figure 1). Based on repeated measurement method analysis the between groups test indicates that the variable group is significant (p = 0.015) and the within subject test indicates that there is not a significant time effect; in other words the groups didn’t change in cytokines over time.

IL-6 was not changed significantly over time and was not different between groups.

### Other Laboratory Tests

In the opium addicted patients levels of ESR (erythrocyte sedimentation rate) and LDL-cholesterol showed higher median levels that were not statistically significant (table 2). There was no significant correlation between laboratory data in the two groups.

## Discussion

Fiotti et al reported that IL-1Ra has a potential role as an acute phase marker. They detected significant increase of IL-1Ra after treadmill test in peripheral arterial disease (PAD) patients [3]. IL-1Ra is a sensitive and reliable indicator of the inflammatory reaction in a chronic condition such as PAD, compared with other indicators of the inflammatory state [3]. They also reported that IL-6 was decreased to undetectable levels following treadmill test [3].

Elevated levels of IL-1 have been described in acute myocardial infarction as soon as 2 hours after symptoms onset and 6 to 9 hours before an increase in IL-6 [5]. Elevated levels of IL-1 have also been described in patients with angina compared with normal subjects. It has been proposed that in atherosclerosis, IL-1 and other inflammatory cytokines are secreted by foam cells (macrophages) in atherosclerotic plaques, as well as by vascular endothelial and smooth muscle cells [13]. Because IL-1Ra is a specific antagonist of IL-1, elevated levels of IL-1Ra could indicate a desirable clinical scenario for reducing the inflammation caused by IL-1 [14]. Though the relative secreted concentrations of IL-1Ra and IL-1β are modulated [15], [16], IL-1Ra is typically produced in much greater abundance. It has been proposed that this agonist/antagonist ratio is an important determinant in the course of inflammatory diseases [17]. Biasucci et al showed that a fall in IL-1Ra and IL-6 after 48 hours in unstable angina was associated with a good outcome, and conversely, an additional increase was associated with a complicated in-hospital course [4]. Also they described elevated levels of IL-6 in unstable angina [5]. In patients with septic shock, a fall in IL-6 levels is associated with survival [18]. Reversible ischemia during Dobutamine stress echocardiography is accompanied by a sustained increase of IL-6 [19]. Interleukin-6 is involved in atherogenesis and plaque destabilization [20] through increased inflammatory stress [19], [20], endothelial dysfunction [21], and local enhancement of thrombosis [22]. Moreover, IL-6 mediates the ischemia reperfusion injury [23], [24] and postischemic impairment of myocardial function [19], [25]. IL-6 is increased in patients with acute coronary syndromes [26] and stable CAD [27], [28] leading to an increased cardiovascular event rate [20], [26], [27], [28], [29]. IL-1 and IL-6 together may contribute to the pathogenesis of acute coronary syndrome [4]. IL-1 is a prototypic proinflammatory cytokine and elicits the production of IL-6, whereas IL-6 does not induce IL-1 production [4], [30].

Neither IL-1Ra nor IL-6 in itself possesses proinflammatory properties [4]. IL-1Ra production increases under the same inflammatory conditions that stimulate IL-1α and IL-1β. IL-1Ra as an anti-inflammatory mediator most precisely correlated with the occurrence and stage of the disease [4]. Patients with atherosclerosis have higher IL-1Ra level [3].

Antiplatelet therapy which in our study administered to nearly all patients was reported not to cause changes in cytokine antagonist/receptor synthesis [31].

In conclusion our results showed significant high IL-1Ra in the addicted patients compared with non-addicted patients that may be related to harm effects of opium in the CAD patients, and stressor maneuver (treadmill test) had no significant effect on this cytokine level. In accordance with other reports, opium not only has any benefit or protective effect in CAD patients, but also is harmful.

The authors suggest further study to compare cytokines levels in the addicted and non-addicted healthy persons and also to conduct this study in a larger group to increase the power of the study.
